# VEGF Promotes Proliferation of Human Glioblastoma Multiforme Stem-Like Cells through VEGF Receptor 2

**DOI:** 10.1155/2013/417413

**Published:** 2013-02-28

**Authors:** Chengshi Xu, Xing Wu, Jianhong Zhu

**Affiliations:** ^1^Department of Neurosurgery, Huashan Hospital, Fudan University, No. 12 Middle Wulumuqi Road, Shanghai 200040, China; ^2^National Key Laboratory for Medical Neurobiology, Institutes of Brain Science, Shanghai Medical College, Fudan University, No. 130 Dong'an Road, Shanghai 200032, China

## Abstract

Cancer stem-like cells, which have been described as tumor-initiating cells or tumor-propagating cells, play a crucial role in our fundamental understanding of glioblastoma multiforme (GBM) and its recurrence. GBM is a lethal cancer, characterized by florid vascularization and aberrantly elevated vascular endothelial growth factor (VEGF). VEGF promotes tumorigenesis and angiogenesis of human GBM stem-like cells (GBSCs). However, whether and how VEGF contributes to GBSCs proliferation remain largely uncertain. In this study, human GBSCs were isolated from surgical specimens of glioblastoma and cultured in medium favored for stem cell growth. Neural Colony-Forming Cell Assay and ATP assay were performed to measure GBSC proliferation under normoxia (20% O_2_) and hypoxia (1% O_2_). Our observations demonstrate that exogenous VEGF stimulates GBSC proliferation in a dose-dependent manner via VEGF Receptor 2 (VEGFR2); while VEGF Receptor 1 (VEGFR1) has a negative feedback effect on VEGFR2 when cells were exposed to higher concentration of VEGF. These results suggest that suppressing VEGFR2-dependent GBSC proliferation is a potentially therapeutic strategy in GBM.

## 1. Introduction

Angiogenesis and tumorigenesis are prominent features of glioblastoma multiforme (GBM). One common thread that connects angiogenesis and tumorigenesis may be vascular endothelial growth factor (VEGF or VEGF-A), which was identified on the basis of its vascular effects [1]. In addition, it has been considered as an important signaling molecule in the nervous system [2, 3].

GBM, the most common primary malignant brain tumor among adults, is characterized by widely spread invasiveness, tumour necrosis, and angiogenesis. Surgical resection, while being effective in removing the primary lesion, cannot remove all of the micrometastases seeded by the migrating glioblastoma cells which, in turn, have been proposed to be glioblastoma stem-like cells (GBSCs) [4, 5]. Thus, genetic, mutational, and proteomic profiling of GBSCs might provide critical indication on the therapeutic targets that may be unique for this small, yet lethal subpopulation of tumor cells.

On the one hand, cancer stem cells are maintained within a special microenvironment, known as niche, which regulates stem cell proliferation and cell-fate decision. GBSCs are indeed maintained within vascular niches that mimic the neural stem cell niche [6]. Endothelial cells may impact the biology of cancer stem cells in the tumor microenvironment by directly interacting with tumor cells [7]. In addition, endothelial cells produce various cytokines, including HGF, VEGF, PDGF, and PIGF. These cytokines stimulate the self-renewal and survival of adjacent cancer stem cells [8, 9]. On the other hand, GBM grows faster than the vasculature, thus leading to an avascular environment deficient of oxygen, leading to hypoxic conditions. Hypoxia can stimulate VEGF secretion through activation of hypoxia-inducible transcription factors (HIFs) [10–12]. VEGF upregulation is associated with a poor response to treatment and poor prognosis. As described previously, VEGF is secreted by endothelial cells, and hypoxia can promote the secretion of VEGF through the HIF pathway. It has been confirmed that the level of VEGF is elevated in GBM, which promotes tumorigenesis and angiogenesis of human GBSCs [13, 14]. Moreover, a number of previous studies have linked VEGF to the proliferation of neural stem cell (NSC) and have shown that GBSCs share some common features with NSC [15, 16]. Hence, VEGF may also play an important role in the survival and proliferation of GBSCs.

VEGF Receptor 1 (VEGFR1, Flt1) and VEGFR2 (KDR/Flk1) are expressed on the cell surface of the human GBSCs. VEGFR2 appears to mediate almost all of the known cellular responses to VEGF. The function of VEGFR1 is to modulate VEGFR2 signaling; also VEGFR1 may act as a decoy receptor, sequestering VEGF from VEGFR2 binding [17, 18].

Collectively, we hypothesize that VEGF can promote the proliferation of GBSCs through VEGFR2 under both normoxic and hypoxic conditions. In order to address this hypothesis, we used a population of stem-like cells derived from patients diagnosed with GBM to study the effect of VEGF on GBSCs proliferation and its related molecular mechanisms.

## 2. Results and Discussion

### 2.1. Glioblastoma Stem-Like Cells Were Derived from Glioblastoma Multiforme

**Figure 1:**
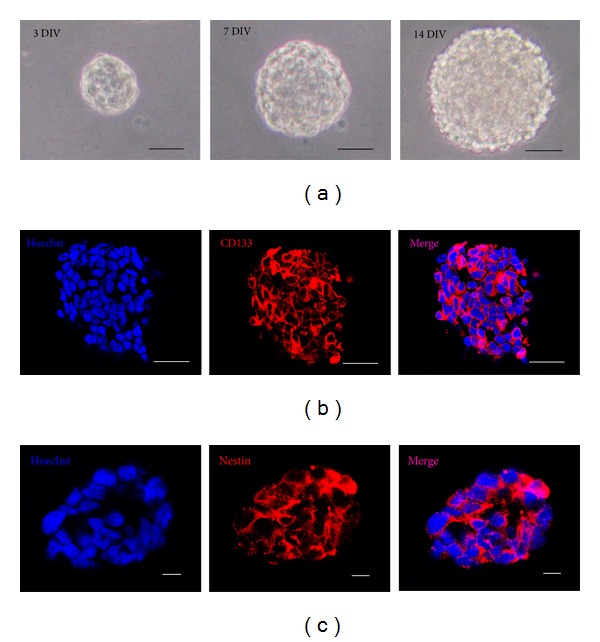
Characterization of GBSCs derived from fresh surgical specimens. (a) Micrograph of GBSCs neurosphere incubated for 3 days, 7 days, and 14 days in vitro (DIV). A single GBSC grown in a define medium can form a neurosphere. Scale bar = 50 *μ*m. (b)-(c) The expression of brain tumor stem cell markers; CD133 and nestin in neurospheres were confirmed by immunofluorescence. Scale bar = 20 *μ*m.

CD133 has been successfully used to enrich putative cancer stem cells. After dissociation of nonadherent tumor spheres, CD133+ cells were identified and enriched by fluorescence-activated cell sorting (FACS). Upon replating at one cell per well, GBSCs spheres formed from single CD133+ cells, usually reaching to a size of 40–60 cells in approximately 2 weeks (Figure 1(a)). Only a small proportion (about 9.6%–11.4%) of the CD133+ tumor cells formed spheres. Sequential minimal dilution assays for at least three passages confirmed that the single-cell-derived tumor spheres had the potential to grow indefinitely. The proportion of sphere-forming cells remained stable throughout the course of culture, indicating GBSCs divided asymmetrically.

GBSCs spheres were found to express brain tumor stem cell markers, CD133 and nestin, which were examined by immunofluorescence (Figures 1(b) and 1(c)).

### 2.2. VEGF Promotes GBSCs Proliferation through VEGFR2 but Not VEGFR1

The effect of VEGF on GBSCs proliferation was tested by Neural Colony-Forming Cell Assay. In addition, to confirm the proliferative phenomenon, which we observed under the microscope, an ATP assay was performed to measure GBSC proliferation.

**Figure 2:**
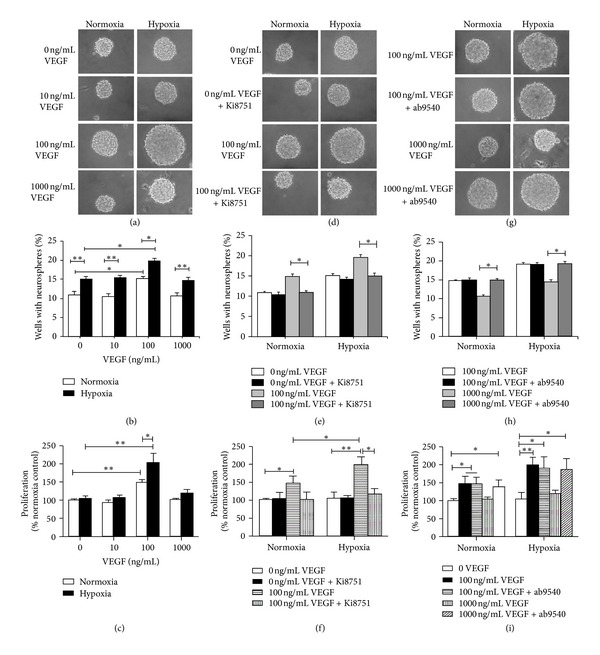
Effect of VEGF on GBSCs proliferation. GBSCs were treated with indicated concentration of VEGF in the presence or absence of VEGFR inhibitors, followed by Neural Colony-Forming Cell Assay and cell viability analysis using ATP assay. (a)–(c) dose effect of VEGF on GBSCs proliferation. (d)–(f) effect of VEGFR2 inhibition on GBSCs proliferation. (g)–(i) effect of VEGFR1 inhibition on GBSCs proliferation. Panels (a), (d), and (g) representative images of neurospheres formed in neurosphere formation assays. Panels (b), (e), and (h) GBSC proliferation was tested using a Neural Colony-Forming Cell Assay; all data are represented as the percent of wells with neurospheres. Panels (c), (f), and (i) GBSCs proliferation was tested using ATP assay; all data are represented as a percentage of the normoxia control. The statistical significance in comparisons between the different groups is indicated using the capped lines (*P < 0.05, **P < 0.01).

As shown in Figures 2(a)–2(c), a significant difference was observed in the percentage of wells with neurospheres under hypoxic condition compared to normoxic condition. Regardless of various concentrations VEGF treatment, hypoxia alone increased proliferation rate of GBSCs. Exogenous VEGF promoted the proliferation of GBSCs in a dose-dependent manner. 100 ng/mL of VEGF significantly increased GBSCs proliferation under both conditions, especially when GBSCs were exposed to hypoxia. However, 10 ng/mL and 1000 ng/mL of VEGF did not show any effect on GBSCs growth under either normoxic or hypoxic conditions. These results demonstrate that an appropriate concentration of VEGF (100 ng/mL) can promote GBSC proliferation and that hypoxic condition has a synergistic effect.

In order to test whether VEGF mediates its action via VEGFR2, the VEGFR2 specific inhibitor (10 nM Ki8751, Selleck) was used in the experiment. Our results showed that Ki8751 did not inhibit GBSCs proliferation when no exogenous VEGF was added. In addition, GBSCs proliferation induced by hypoxia alone could not be suppressed by Ki8751, indicating that hypoxia might promote GBSCs proliferation via other signalling pathways. However, when GBSCs were cultured with 100 ng/mL VEGF, VEGFR2 inhibitor dramatically decreased GBSC proliferation (Figures 2(d)–2(f)) under both conditions, indicating that VEGFR2 mediates VEGF-induced cell proliferation in GBSCs.

We further tested the function of VEGFR1 on GBSC proliferation using an antibody directed against this protein (1 : 100 ab9540, Abcam). Consistent with the neurosphere formation assay, 1000 ng/mL of VEGF significantly decreased GBSC growth compared with the 100 ng/mL VEGF-treated group; however, this effect was reversed by adding a VEGFR1 monoclonal antibody under both normoxia and hypoxia, which was in parallel to the 100 ng/mL VEGF-treated group (Figures 2(g)–2(i)), indicating that VEGFR1 may have a negative feedback effect on cell proliferation when the cells were exposed to a higher concentration of VEGF.

### 2.3. VEGFR2 Signaling but Not VEGFR1 Signaling Is Involved in GBSCs Proliferation

**Figure 3:**
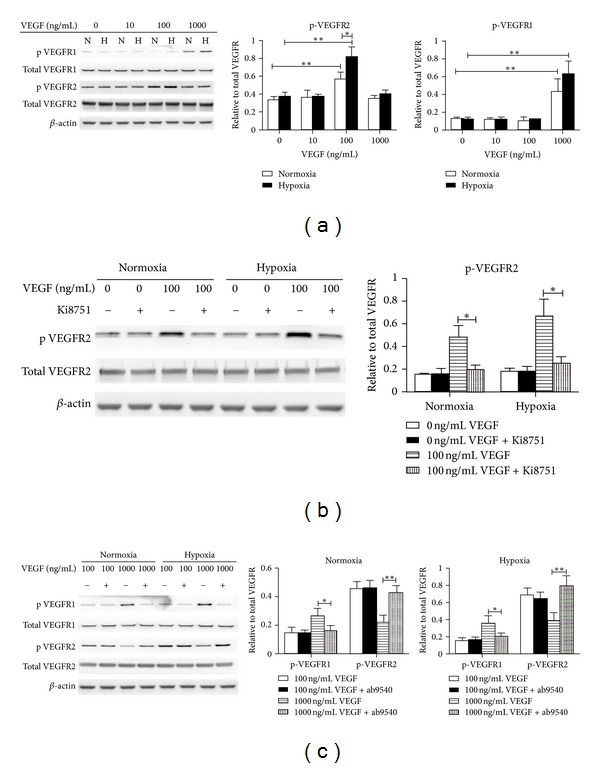
Effect of VEGF on VEGFR expression. GBSCs were treated with the indicated concentration of VEGF in the presence or absence of the VEGFR inhibitor or antibody. The levels of total VEGFR1 and VEGFR2 protein and the levels of phosphorylated VEGFR1 and VEGFR2 were determined by Western blot analysis and expressed relative to *β*-actin protein level. Phosphorylated VEGFR1 and VEGFR2 protein levels were normalized to total VEGFR1 and VEGFR2, respectively. Values represent the mean ± SD of three separate experiments. The statistical significance in comparisons between the different groups is indicated using the capped lines (N = normoxia, H = hypoxia;*P < 0.05, **P < 0.01).

To test how GBSCs proliferation is coupled with VEGFR1 and/or VEGFR2 signaling, we next analyzed the total and phosphorylated VEGFR1 and VEGFR2 protein expressions in GBSCs after VEGF treatment. In line with our expectations, 100 ng/mL of VEGF significantly increased the phosphorylated protein level of VEGFR2 under both normoxic and hypoxic conditions; and the phosphorylation level increased even more when GBSCs were exposed to hypoxia (Figure 3(a)). However, the level of phosphorylated VEGFR1 showed no significant difference compared with the control group, indicating that VEGFR2 signaling but not VEGFR1 signaling is involved in GBSCs proliferation. Interestingly, compared to 100 ng/mL VEGF-treated group, 1000 ng/mL of VEGF significantly increased the phosphorylated protein level of VEGFR1 but reduced phosphorylated VEGFR2. Furthermore, the phosphorylated VEGFR2 did not increase when GBSCs were exposed to hypoxia, which is consistent with GBSCs proliferation induced by hypoxia alone.

VEGFR2 inhibitor but not VEGFR1 monoclonal antibody blocked the increase in VEGFR2 phosphorylation level, and VEGFR2 inhibitor did not alter the phosphorylation level of VEGFR1 (data not show) (Figure 3(b)). Contrary to VEGFR2 inhibitor, VEGFR1 antibody increased the phosphorylated level of VEGFR2 when GBSCs were treated with high concentration of VEGF, whereas VEGFR1 antibody did not enhance the VEGFR2 phosphorylation level when VEGF concentration was decreased to 100 ng/mL (Figure 3(c)), indicating that VEGFR1 has a negative feedback effect on VEGFR2 when cells were exposed to higher concentration of VEGF.

### 2.4. Discussion

Recent evidence suggests that GBSCs may be important for the initiation, propagation, and recurrence of glioblastoma, and hence GBSCs are now emerging as critical therapeutic targets [19]. Therefore, understanding of the molecular mechanisms involved in regulating biological behavior of GBSCs may be a significant matter. Previous studies showed that endothelial-derived factors accelerate the initiation and growth of tumors in the brain, highlighting the specificity of the function relationship between endothelial cells and brain tumor stem cells [8]. However, it remains unclear how these growth factors interact with each other. To answer this question, first of all, we should understand the effects of VEGF, which may be the most significant endothelial-derived factor, in regulating GBSC proliferation.

The concept emerging from our study can be summarized as follows: (1) irrespective of normoxic or hypoxic conditions, exogenous VEGF stimulates GBSC proliferation in a dose-dependent manner. At the dosage of 100 ng/mL, VEGF promotes GBSCs proliferation; however, at high dosages, for instance, 1000 ng/mL, it does not show the same effect. (2) VEGF enhances GBSC proliferation through VEGFR2; Ki8751, a VEGFR2-specific inhibitor, suppresses VEGF-induced GBSC proliferation. (3) VEGFR1 downregulates the positive effect of VEGF in the proliferation of GBSCs, and the effect of VEGFR1 is inhibited by an antibody raised against. The biological outcome of stimulating or inhibiting the activity of VEGFR1 might be influenced through “crosstalk” with VEGFR2. Several groups have reported that VEGFR1 negatively regulates VEGFR2 signals [20, 21]. Moreover, embryonic stem cells that lack VEGFR1 show increased levels of VEGFR2 phosphorylation [20]. It has been shown that an alternately spliced form of VEGFR1 (FLT1) produces a soluble protein, known as sFLT1, which binds to and has a high affinity to VEGF. Because sFLT1 has a higher affinity to VEGF than does VEGFR2, it may act as an inhibitor of VEGF response [22]. (4) VEGF coordinates with hypoxia in regulating GBSCs proliferation; however, the effect of hypoxia in promoting of GBSCs proliferation is independent on the presence of VEGF; VEGFR2 inhibitor could not suppress hypoxia-induced GBSCs proliferation. The reason for this may be that under hypoxic condition, GBSCs also secret many other growth factors. These findings are consistent with Bao et al, who showed that high level of VEGF, produced by CD133+ human glioblastoma cells, might contribute to their tumour-initiating capacity [23]. This novel finding enhances our understanding on the mechanism by which VEGF in regulating GBSCs proliferation and may provide insight into the regulation of GBSC by VEGF signaling.

The prognosis for GBM is very poor, and novel treatment strategies are urgently needed. GBM is a highly vascular tumor; a result of its increased expression of VEGF during progression is compared with other brain tumors [24, 25]. Increased levels of VEGF in GBM accelerate vascular proliferation and exacerbate the disease [26]. Based on our present data, we propose that VEGF may be the dual targets of not only the tumor vessels but also the tumor stem cells. Multiple treatment modalities have targeted VEGF and VEGFRs due to their significant roles in regulating angiogenic processes and GBSCs proliferation. In glioma patients, anti-VEGF and VEGFR2 inhibitors are commonly used to target the VEGF-VEGFR2 signaling cascade. However, despite some transient positive therapeutic effects, the efficacy of these two strategies has been disappointing [27–31]. Antiangiogenic therapy leads to devascularization that limits tumor growth, but the benefits of angiogenesis inhibitors are typically transient, and resistance often develops [27]. In addition, some antiangiogenic agents have been shown to promote tumor growth and metastasis [29]. Multiple mechanisms may be involved, and induction of hypoxia which promotes GBSC proliferation might be one of the these mechanisms [29, 32]. Collectively, our results demonstrate that hypoxia can stimulate GBSC proliferation, which cannot be suppressed by VEGFR inhibitors. We believe that not only VEGF but also some other growth factor signaling contributes to the enhanced GBSC proliferation under a hypoxic condition, but VEGF is not essential. With prolonged antiangiogenic treatment, tumors develop progressive hypoxia, which may be a central factor in promoting tumor resistance to therapy and ultimately tumor progression. The strong correlation between GBSC proliferation and the level of hypoxia further supports the idea that hypoxia is an important initiator of tumor growth and metastasis.

In summary, our results have many implications for clinical practice. VEGF plays a positive role in regulating angiogenesis and tumorigenesis; as a result, VEGF inhibition is a potential effective treatment strategy in GBM. Our findings emphasize the requirement to target pathways involved in the development of resistance to antiangiogenic treatment, such as hypoxia.

## 3. Experimental Section

### 3.1. GBM Tissues

Surgical specimens of GBM were obtained after patients' written consent under a protocol approved by the institution's Institutional Review Board. The neuropathological review of gliomas was completed by a neuropathology specialist.

### 3.2. Isolation of Glioma Stem-Like Cells

GBSCs were isolated from primary human brain tumor patient specimens. Fresh surgical specimens were obtained from 12 patients diagnosed with glioblastoma multiforme (GBM). Briefly, tumor tissues were washed and minced with fine scissors into small fragments, which were disaggregated by Papain Dissociation System and filtered by 70 *μ*m cell strainer according to the manufacturer's instructions. Cells were then cultured in stem cell culture medium for at least 4 hours to recover surface antigens. Cells were then labeled with APC- or PE-conjugated CD133 antibody and sorted by fluorescence-activated cell sorting (FACS) as described previously [33]. CD133-positive cells were designated as GBSCs, which were resuspended in serum-free DMEM/F-12 containing human recombinant N_2_ (20 ng/mL; Invitrogen), EGF (20 ng/mL; Invitrogen), and bFGF (20 ng/mL; Gibco) and then seeded in a nonadherent cell culture flask (5 mL per flask) at a density of 5 × 10^6^ live cells per flask. Culture media was changed twice a week, and neurospheres are passaged every 1 or 2 weeks, depending on the growth rate of each sample. Immunofluorescence staining to detect the expression of brain tumor stem cell markers was performed as described previously [33].

### 3.3. Neural Colony-Forming Cell Assay

Neurosphere formation assays were performed with CD133-positive cells sorted by FACS to single cells per well of 96 well plates in a collagen semisolid matrix (Stem Cell Technologies). The semisolid matrix contained serum-free media supplemented with human recombinant N_2_ (20 ng/mL; Invitrogen), EGF (20 ng/mL; Invitrogen), and bFGF (20 ng/mL; Gibco). Various concentrations of VEGF (0 ng/mL, 10 ng/mL, 100 ng/mL, and 1000 ng/mL; Sigma) in the presence or absence of VEGFR inhibitors were added as previously described, and cells were incubated for 14 days in vitro (DIV) under normoxic (20% O_2_) or hypoxic (1% O_2_) condition. In order to induce hypoxia, cells were cultured in hypoxia chambers (Sanyo). Complete replenishment medium was added into the center of each NCFC dish once every 3 days during the entire NCFC culture incubation (14 days). Neurosphere formation was measured as the percent of wells with neurospheres after 14 days.

### 3.4. ATP Assay

Primary neurospheres were dispersed with 0.025% trypsin and mechanical trituration after trypsin inactivation. 10,000 single GBSCs diluted in 100 *μ*L of medium were seeded in a 96-well plate and cultured for 24 hrs. The cells were then treated with indicated concentrations of VEGF in the presence or absence of VEGFR inhibitors under normoxic (20% O_2_) and hypoxic (1% O_2_) conditions. After 24 hrs of incubation, cells were equilibrated at room temperature for 30 min, and then 100 *μ*L of ATP reagent (CellTiter-Glo luminescent cell viability assay, Promega) was added to each well. Cell lysis was induced on an orbital shaker for 2 min, then equilibrated at room temperature for 10 min, and the luminescent signal was recorded in a PolarStar plate reader.

### 3.5. Western Blotting

For Western blot analysis, cells grown in 6-well plate to confluence were treated as described in the section on the ATP assay. Cells floating in medium were then harvested, washed twice with ice-cold PBS, and lysed in RIPA buffer (Sigma) with freshly added protease and phosphatase inhibitor cocktail (Thermo Scientific). Cells were allowed to lyse for 10 min on ice and centrifuged at 12,000 g for 15 min at 4°C. The supernatant was removed, and protein concentrations in the supernatant were determined by BCA protein assay. A total of 40 *μ*g of proteins were loaded on 4–15% SDS polyacrylamide gradient gels and transferred onto a NC membrane. The membrane was sequentially incubated with a 5% fat-free milk for 1 hour at room temperature, a specific primary antibody for total and phosphorylated VEGFR1 and VEGFR2 (cell signaling) overnight at 4°C and appropriated HRP-conjugated second antibody for 1 hour at room temperature. Immunoreactive bands were detected using the ECL chemiluminescence reagent (GE Healthcare-Amersham Biosciences). The membrane was stripped using stripping buffer (Amersham Biosciences) and subsequently labeled with *β*-actin following the standard Western blot procedures. Densitometry was analyzed using Quantity One software (Bio-Rad). Densitometry results were either normalized to total protein or *β*-actin.

### 3.6. Statistical Analysis

All experiments were repeated at least three times. The statistical significance of differences was evaluated by a one-way analysis of variance (ANOVA) or Student's *t*-test (Prism 5; GraphPad Software, San Diego, http://www.graphpad.com/). Descriptive statistics were generated for all quantitative data with presentation of means ± standard error. The level of significance for all comparisons was *P* < 0.05.

## 4. Conclusions

VEGF stimulates GBSC proliferation in a dose-dependent manner via VEGFR2 signaling, and VEGFR1 has a negative feedback effect on VEGFR2. This novel finding enhances our understanding of the mechanism by which VEGF regulates GBSC proliferation. In addition, Our results support the hypothesis that the devascularization caused by anti-VEGF therapy increases tumor hypoxia, and this hypoxia mediates resistance to antiangiogenic therapy.

## References

[1] Greenberg DA, Jin K (2005). From angiogenesis to neuropathology. *Nature*.

[2] Mackenzie F, Ruhrberg C (2012). Diverse roles for VEGF-A in the nervous system. *Development*.

[3] Rosenstein JM, Krum JM (2004). New roles for VEGF in nervous tissue—beyond blood vessels. *Experimental Neurology*.

[4] Cheshier SH, Kalani MYS, Lim M, Ailles L, Huhn SL, Weissman IL (2009). A neurosurgeon’s guide to stem cells, cancer stem cells, and brain tumor stem cells. *Neurosurgery*.

[5] Piccirillo SGM, Binda E, Fiocco R, Vescovi AL, Shah K (2009). Brain cancer stem cells. *Journal of Molecular Medicine*.

[6] Takakura N (2012). Formation and regulation of the cancer stem cell niche. *Cancer Science*.

[7] Zhu TS, Costello MA, Talsma CE (2011). Endothelial cells create a stem cell niche in glioblastoma by providing notch ligands that nurture self-renewal of cancer stem-like cells. *Cancer Research*.

[8] Hamerlik P, Lathia JD, Rasmussen R (2012). Autocrine VEGF-VEGFR2-neuropilin-1 signaling promotes glioma stem-like cell viability and tumor growth. *The Journal of Experimental Medicine*.

[9] Jinushi M, Baghdadi M, Chiba S, Yoshiyama H (2012). Regulation of cancer stem cell activities by tumor-associated macrophages. *American Journal of Cancer Research*.

[10] Heddleston JM, Li Z, Lathia JD, Bao S, Hjelmeland AB, Rich JN (2010). Hypoxia inducible factors in cancer stem cells. *British Journal of Cancer*.

[11] Keith B, Simon MC (2007). Hypoxia-inducible factors, stem cells, and cancer. *Cell*.

[12] Li Z, Bao S, Wu Q (2009). Hypoxia-inducible factors regulate tumorigenic capacity of glioma stem cells. *Cancer Cell*.

[13] Folkins C, Shaked Y, Man S (2009). Glioma tumor stem-like cells promote tumor angiogenesis and vasculogenesis via vascular endothelial growth factor and stromal-derived factor 1. *Cancer Research*.

[14] Mentlein R, Forstreuter F, Mehdorn HM, Held-Feindt J (2004). Functional significance of vascular endothelial growth factor receptor expression on human glioma cells. *Journal of Neuro-Oncology*.

[15] Xiao Z, Kong Y, Yang S, Li M, Wen J, Li L (2007). Upregulation of Flk-1 by bFGF via the ERK pathway is essential for VEGF-mediated promotion of neural stem cell proliferation. *Cell Research*.

[16] Zhao D, Najbauer J, Garcia E (2008). Neural stem cell tropism to glioma: critical role of tumor hypoxia. *Molecular Cancer Research*.

[17] Bruns AF, Bao L, Walker JH, Ponnambalam S (2009). VEGF-A-stimulated signalling in endothelial cells via a dual receptor tyrosine kinase system is dependent on co-ordinated trafficking and proteolysis. *Biochemical Society Transactions*.

[18] Olsson AK, Dimberg A, Kreuger J, Claesson-Welsh L (2006). VEGF receptor signalling—in control of vascular function. *Nature Reviews Molecular Cell Biology*.

[19] Hadjipanayis CG, Van Meir EG (2009). Brain cancer propagating cells: biology, genetics and targeted therapies. *Trends in Molecular Medicine*.

[20] Roberts DM, Kearney JB, Johnson JH, Rosenberg MP, Kumar R, Bautch VL (2004). The vascular endothelial growth factor (VEGF) receptor flt-1 (VEGFR-1) modulates flk-1 (VEGFR-2) signaling during blood vessel formation. *American Journal of Pathology*.

[21] Kappas NC, Zeng G, Chappell JC (2008). The VEGF receptor Flt-1 spatially modulates Flk-1 signaling and blood vessel branching. *Journal of Cell Biology*.

[22] Sela S, Natanson-Yaron S, Zcharia E, Vlodavsky I, Yagel S, Keshet E (2011). Local retention versus systemic release of soluble VEGF receptor-1 are mediated by heparin-binding and regulated by heparanase. *Circulation Research*.

[23] Bao S, Wu Q, Sathornsumetee S (2006). Stem cell-like glioma cells promote tumor angiogenesis through vascular endothelial growth factor. *Cancer Research*.

[24] Linkous AG, Yazlovitskaya EM (2011). Angiogenesis in glioblastoma multiforme: navigating the maze. *Anti-Cancer Agents in Medicinal Chemistry*.

[25] Robles Irizarry L, Hambardzumyan D, Nakano I, Gladson CL, Ahluwalia MS (2012). Therapeutic targeting of VEGF in the treatment of glioblastoma. *Expert Opinion on Therapeutic Targets*.

[26] Chi AS, Sorensen AG, Jain RK, Batchelor TT (2009). Angiogenesis as a therapeutic target in malignant gliomas. *Oncologist*.

[27] Hu YL, DeLay M, Jahangiri A (2012). Hypoxia-induced autophagy promotes tumor cell survival and adaptation to antiangiogenic treatment in glioblastoma. *Cancer Research*.

[28] Bikfalvi A, Moenner M, Javerzat S, North S, Hagedorn M (2011). Inhibition of angiogenesis and the angiogenesis/invasion shift. *Biochemical Society Transactions*.

[29] Keunen O, Johansson M, Oudin A (2011). Anti-VEGF treatment reduces blood supply and increases tumor cell invasion in glioblastoma. * Proceedings of the National Academy of Sciences of the United States of America*.

[30] Norden AD, Drappatz J, Wen PY (2009). Antiangiogenic therapies for high-grade glioma. *Nature Reviews Neurology*.

[31] Chamberlain MC (2008). Antiangiogenic blockage: a new treatment for glioblastoma. *Expert Opinion on Biological Therapy*.

[32] Heddleston JM, Wu Q, Rivera M (2012). Hypoxia-induced mixed-lineage leukemia 1 regulates glioma stem cell tumorigenic potential. *Cell Death & Differentiation*.

[33] Tang H, Gong Y, Mao Y (2012). Cd133-positive cells might be responsible for efficient proliferation of human meningioma cells. *International Journal of Molecular Sciences*.

